# Prothrombotic effects of high uric acid in mice *via* activation of MEF2C-dependent NF-κB pathway by upregulating let-7c

**DOI:** 10.18632/aging.103540

**Published:** 2020-09-22

**Authors:** Xiaoyu Cheng, Tian Liu, Lidan Ma, Zhen Liu, Ying Xin, Zhaotong Jia, Ying Chen, Changgui Li, Ruixia Sun

**Affiliations:** 1 Department of Endocrinology and Metabolism, The Affiliated Hospital of Qingdao University, Qingdao 266003, P.R. China

**Keywords:** thrombosis, high uric acid, let-7c, myocyte enhancer factor-2C, nuclear factor-kappa B pathway

## Abstract

Serum uric acid is reportedly associated with thrombosis development. However, still unclear is the mechanism of high uric acid in thrombosis with the involvement of let-7c. In an aim to fill this void, we conducted this study by treating mice and human umbilical vein endothelial cells with high uric acid. Analysis indicated that let-7c was upregulated in hyperuricemia patients as well as in mice and human umbilical vein endothelial cells treated with high uric acid. Furthermore, high uric acid inhibited myocyte enhancer factor-2C, but activated nuclear factor-kappa B pathway in human umbilical vein endothelial cells. Then the targeting relationship between let-7c and myocyte enhancer factor-2C was verified. On the one hand, high uric acid shortened activated partial thromboplastin time and prothrombin time of mice and declined tissue plasminogen activator level. Additionally, the treatment prolonged thrombin time and elevated the levels of thrombosis related molecules or proteins such as Fibrinogen and D-dimer. Nevertheless, these alternations could be reversed by inhibition of let-7c and nuclear factor-kappa B pathway or overexpressing myocyte enhancer factor-2C. To sum up, our results uncovered the pro-thrombotic effect of high uric acid in mice by activating myocyte enhancer factor-2C-dependent nuclear factor-kappa B pathway *via* let-7c upregulation.

## INTRODUCTION

As a principle cause of fatality worldwide, thrombosis has a close relationship with hemostasis, in which blood coagulation and platelet activation play pivotal roles [1]. Thrombosis is the most common pathology leading to venous thromboembolism (VTE), ischemic heart disease, and ischemic stroke [2]. Furthermore, thrombosis is a major complication of chronic inflammatory conditions such as obesity, and inflammatory bowel disease or other autoimmune disorders [3]. The mediation of thrombosis depends on mechanics, hydrodynamics and mass transport of various species, blood cell adhesion, spatiotemporal regulation of the blood coagulation network, and complex signal transduction networks in platelets [4]. It was identified that uric acid was higher in Behcet’s disease patients with thrombosis than in those without thrombotic complication [5], suggesting a relationship between high uric acid (HUA) and thrombosis. As an organic compound, uric acid is a purine metabolite endogenously produced by animals, and HUA is a precipitating factor for cardiovascular disease [6]. Therefore, thrombosis treatment would benefit a better understanding of the molecular mechanism of HUA in thrombosis.

Moreover, a prior study revealed that HUA contributes to the upregulation of lethal-7c (let-7c) [7]. Let-7 is the second miRNA family found *in Caenorhabditis elegans* and is also the first in humans, which consists of 13 members distributed across nine chromosomes [8]. Moreover, the alteration of let-7c is involved in the regulation of platelets [9]. Other preclinical research showed that let-7 promoted the development of myocardial infarction induced by permanent ligation of the left anterior descending coronary artery [10]. The targetscan website predicted that myocyte enhancer factor-2C (MEF2C) is a target gene of let-7c. MEF2C, a transcription factor of MADS box family, participates in the early development of many human cells, including muscle (i.e., bone, heart and smooth muscle), nerve, cartilage, immune, and endothelial cells [11]. A recent study suggested that MEF2C can inhibit coagulation and improve arteriosclerosis [12]. Additionally, MEF2C inactivates nuclear factor-kappa B (NF-κB) to inhibit endothelial cell inflammation [13]. Meanwhile, NF-κB activation occurs in human renal tubular epithelial cells (HK-2) treated with HUA [14]. More importantly, cancer patients with deep vein thrombosis (DVT) had high expression of NF-κB [15]. Based on the aforesaid literature, we hypothesized that let-7c was involved in the functional mechanism of HUA in thrombosis *via* MEF2C and NF-κB pathway. To test this hypothesis, we generated a hyperuricemia mouse model through high yeast feeding to explore the effects of HUA and let-7c on thrombosis.

## RESULTS

### Upregulation of let-7c is observed in serum of patients with hyperuricemia

**Figure 1:**
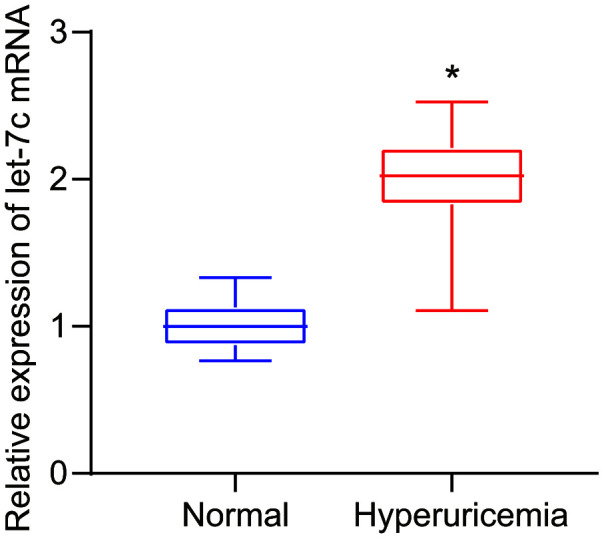
**Let-7c is upregulated in serum of patients with hyperuricemia, as determined by RT-qPCR (normalized to U6).** n = 26. * *p* < 0.05 *vs.* healthy people. The measurement data were shown as mean ± standard deviation and compared by unpaired *t*-test.

As prior literature indicates, HUA stimulates let-7c expression in human umbilical vein endothelial cells (HUVECs) [7]. To further verify whether HUA affected hyperuricemia *via* let-7c, let-7c expression was determined in serum of patients with hyperuricemia. As presented in Figure 1, high let-7c expression appeared in serum of patients with hyperuricemia. Therefore, let-7c upregulation correlates to hyperuricemia development.

### HUA promotes hemagglutination and thrombosis by upregulating let-7c in mice

**Figure 2:**
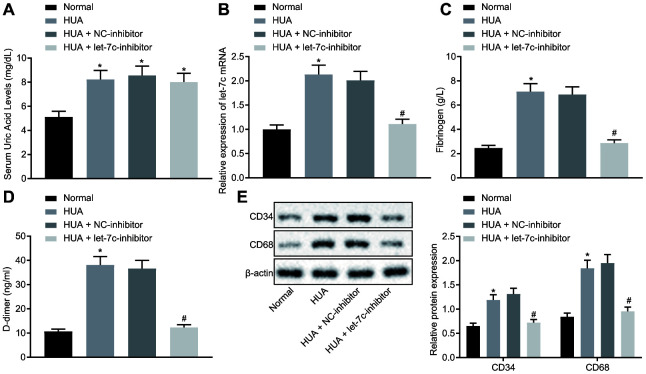
**HUA increases let-7c expression to stimulate hemagglutination and thrombosis in mice.** Normal mice were used as controls, and HUA mice were treated or not treated with NC-inhibitor or let-7c-inhibitor. (**A**) SUA in serum of mice detected using Urea Assay Kit. (**B**) Let-7c expression in serum of mice detected by RT-qPCR normalized to U6. (**C**) Fibrinogen level in serum of mice determined by ELISA. (**D**) D-dimer level in serum of mice determined by ELISA. (**E**) western blot analysis of CD36 and CD41 in platelets of mice normalized to β-actin. * *p* < 0.05 *vs.* normal mice. # *p* < 0.05 *vs.* HUA mice treated with NC-inhibitor. The measurement data were shown as mean ± standard deviation and compared by one-way analysis of variance, followed by Tukey's post hoc test. n = 10.

To verify the effect of HUA on hemagglutination and thrombosis *in* *vivo*, mice were treated with HUA to induce hyperuricemia. As depicted in Figure 2A, the SUA level was higher in serum of HUA mice than in normal mice (*p* < 0.05). Then, reverse transcription quantitative polymerase chain reaction (RT-qPCR) revealed that let-7c expression was increased in serum of mice after HUA treatment and decreased in serum of HUA mice following let-7c-inhibitor treatment (Figure 2B). Then, coagulation parameters, including activated partial thromboplastin time (APTT), prothrombin time (PT) and thrombin time (TT), were measured in serum of the mice. The data displayed shortened APTT and PT and prolonged TT in HUA mice, but these alterations could be reversed by treatment with a let-7c-inhibitor (Table 1). Enzyme-linked immunosorbent assay (ELISA) documented that fibrinogen and D-dimer levels were enhanced in serum of HUA mice, and that let-7c-inhibitor reversed these effects in HUA mice (Figure 2C, 2D). Moreover, after HUA treatment, CD36 and CD41 were also significantly elevated, but this effect was reversed following let-7c-inhibitor treatment (Figure 2E). Taken together, let-7c promotes by HUA induced hemagglutination and thrombosis in mice.

**Table 1:** Levels of APTT, PT and TT in serum of mice.

**Groups**	**APTT/s**	**PT/s**	**TT/s**
Normal	26.19 ± 2.38	9.06 ± 0.82	18.09 ± 1.64
HUA	8.69 ± 0.79*	4.26 ± 0.39*	30.06 ± 2.73*
HUA + NC-inhibitor	9.22 ± 0.84	4.91 ± 0.45	28.77 ± 2.62
HUA + let-7c-inhibitor	25.11 ± 2.28#	8.87 ± 0.81#	19.22 ± 1.75#

Note: * *p* < 0.05 *vs.* normal mice. # *p* < 0.05 *vs.* HUA mice treated with NC-inhibitor. The measurement data were shown as mean ± standard devia.

### HUA increases the expression of thrombus-related factors and the adhesion of monocytes and platelets *via* let-7c upregulation

**Figure 3:**
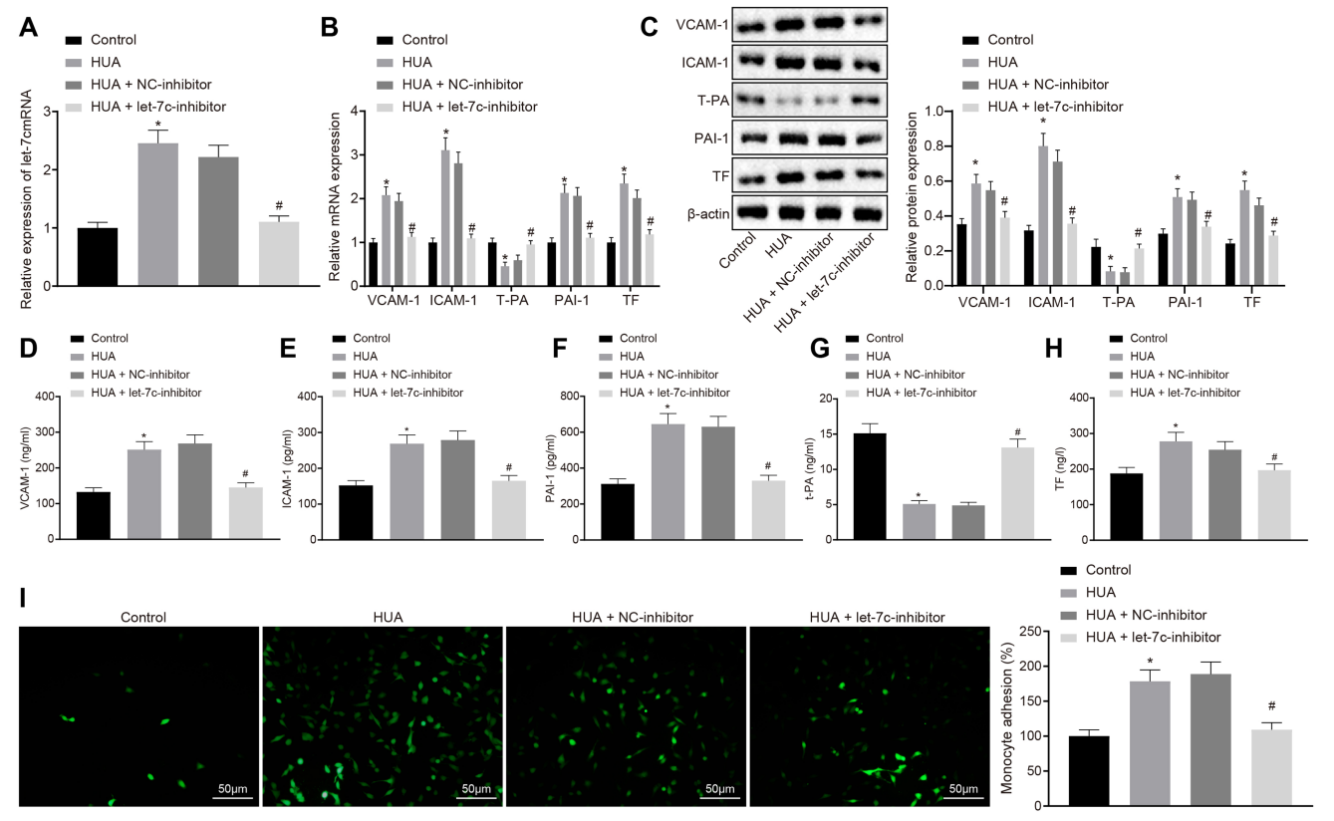
**HUA-activated let-7c increases the expression of thrombus-related factors and the adhesion of monocytes and platelets to HUVECs. Normal HUVECs were used as controls, and HUA HUVECs were treated or not treated with NC-inhibitor or let-7c-inhibitor.** (**A**) Let-7c expression in HUVECs. (**B**) VCAM-1, ICAM-1, PAI-1, TF, and T-PA expression in HUVECs determined by RT-qPCR normalized to GAPDH. (**C**) VCAM-1, ICAM-1, PAI-1, TF and T-PA expression in HUVECs determined by western blot analysis normalized to β-actin. (**D**) VCAM-1 level in HUVECs determined by ELISA. (**E**) ICAM-1 level in HUVECs determined by ELISA. (**F**) PAI-1 level in HUVECs determined by ELISA. (**G**) TF level in HUVECs determined by ELISA. (**H**) T-PA level in HUVECs determined by ELISA. (**I**) Adhesion of monocytes to HUVECs (× 200). * *p* < 0.05 *vs.* control HUVECs. # *p* < 0.05 *vs.* HUA HUVECs treated with NC-inhibitor. The measurement data were shown as mean ± standard deviation and compared by one-way analysis of variance, followed by Tukey's post hoc test. The cell experiment was repeated three times independently.

To study further whether HUA impairs the function of HUVECs by promoting let-7c expression, we treated HUVECs with HUA, and applied treatment with let-7c-inhibitor. RT-qPCR (Figure 3A, 3B), western blot analysis (Figure 3C) and ELISA (Figure 3D–3H) demonstrated that, following HUA treatment, let-7c, vascular cell adhesion protein 1 (VCAM-1), interstitial cell adhesion molecule (ICAM)-1, plasminogen activator inhibitor (PAI)-1 and tissue factor (TF) protein expression were obviously enhanced, whereas tissue plasminogen activator (T-PA) expression was potently decreased in HUVECs; these effects were abolished by let-7c-inhibitor. As described in Figure 3I, adhesion of monocytes to HUVECs was increased substantially by HUA treatment, which was neutralized by let-7c-inhibitor. These results suggest that the expression of thrombus-related factors and the adhesion of monocytes and platelets to HUVECs are promoted by HUA-upregulated let-7c.

### Let-7c negatively targets MEF2C

**Figure 4:**
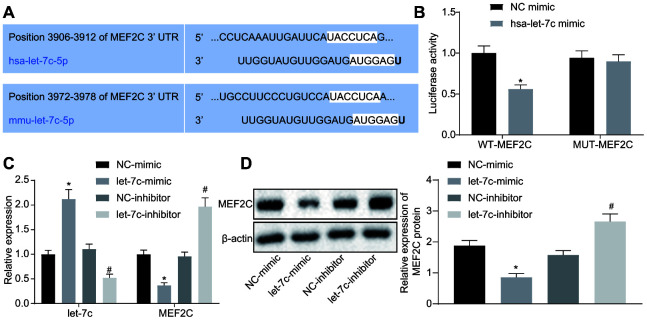
**MEF2C is negatively targeted by let-7c.** (**A**) The binding sites between let-7c and MEF2C predicted in targetscan. (**B**) The binding relationship between let-7c and MEF2C evaluated by dual luciferase reporter assay. * *p* < 0.05 *vs.* the treatment of NC-mimic. (**C**) Expression of let-7c and MEF2C in HUA-treated HUVECs after alteration of let-7c determined by RT-qPCR normalized to U6 and GAPDH. (**D**) Protein expression of MEF2C in HUA-treated HUVECs after alteration of let-7c determined by western blot analysis normalized to β-actin. * *p* < 0.05 *vs.* HUA HUVECs treated with NC-mimic. # *p* < 0.05 *vs.* HUA HUVECs treated with NC-inhibitor. * *p* < 0.05 *vs.* normal HUVECs. The measurement data were shown as mean ± standard deviation. Data between two groups were compared by unpaired t-test, while comparisons among multiple groups were performed using one-way analysis of variance, followed by Tukey's post hoc test. The cell experiment was repeated three times independently.

The focus of the study was then shifted to the downstream mechanism of let-7c in thrombosis. First, the binding sites between let-7c and MEF2C were predicted by targetscan (Figure 4A). Moreover, dual luciferase reporter assay revealed that the luciferase activity was reduced obviously in wild type (WT)-MEF2C after let-7c-mimic treatment, but was unchanged in mutation (MUT)-MEF2C (Figure 4B), indicating a targeting relationship between let-7c and MEF2C. Furthermore, in HUVECs, treatment with let-7c-mimic reduced, but let-7c-inhibitor elevated the mRNA (Figure 4C) and protein (Figure 4D) expression of MEF2C. Taken together, MEF2C is confirmed as a target gene of let-7c.

### HUA-activated let-7c promotes the expression of thrombus-related factors and the adhesion of monocytes and platelets to HUVECs by downregulating MEF2C

**Figure 5:**
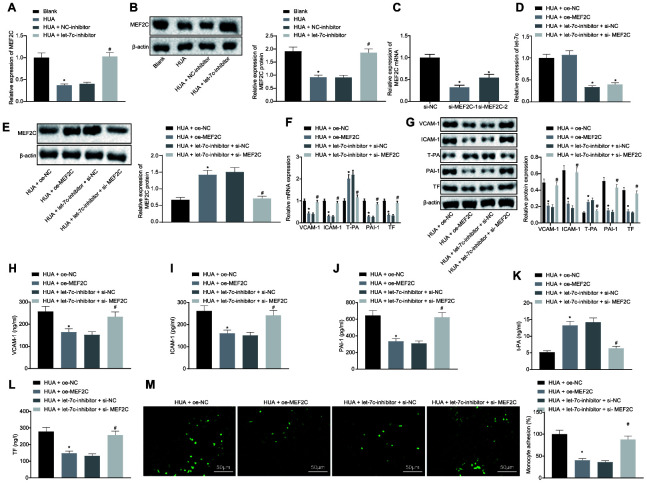
**The expression of thrombus-related factors and the adhesion of monocytes and platelets to HUVECs are promoted by HUA *via* let-7c-downregulated MEF2C.** (**A**) RT-qPCR analysis of MEF2C expression in HUVECs after treatment with HUA. (**B**) western blot analysis of MEF2C protein expression in HUVECs. (**C**) si-MEF2C-1 and si-MEF2C-2 detected by RT-qPCR normalized to GAPDH. HUA-treated HUVECs were transfected with oe-NC, oe-MEF2C, let-7c-inhibitor + si-NC or let-7c-inhibitor + si-MEF2C. (**D**) Let-7c expression in HUVECs detected by RT-qPCR normalized to U6. (**E**) Protein expression of MEF2C in HUVECs detected by western blot analysis normalized to β-actin. (**F**) VCAM-1, ICAM-1, PAI-1, TF and T-PA expression in HUVECs determined by RT-qPCR normalized to GAPDH. (**G**) VCAM-1, ICAM-1, PAI-1, TF and T-PA expression in HUVECs determined by western blot analysis normalized to β-actin. (**H**) VCAM-1 level in HUVECs determined by ELISA. (**I**) ICAM-1 level in HUVECs determined by ELISA. (**J**) PAI-1 level in HUVECs determined by ELISA. (**K**) TF level in HUVECs determined by ELISA. (**L**) T-PA level in HUVECs determined by ELISA. (**M**) Adhesion of monocytes to HUVECs (× 200). * *p* < 0.05 *vs.* HUA HUVECs transfected with oe-NC. # *p* < 0.05 *vs.* HUA HUVECs transfected with let-7c-inhibitor + si-NC. The measurement data were shown as mean ± standard deviation and compared by one-way analysis of variance, followed by Tukey's post hoc test. The cell experiment was repeated three times independently.

Subsequently, HUVECs were treated with HUA and let-7c-inhibitor to investigate further whether HUA could downregulate MEF2C by activating let-7c to affect the function of HUVECs. RT-qPCR and western blot analysis revealed that HUA treatment triggered decline of MEF2C expression in HUVECs, which was restored by let-7c-inhibitor (Figure 5A, 5B). To study further the function of MEF2C, two MEF2C silencing sequences, si-MEF2C-1 and si-MEF2C-2, were designed. After treatment with si-MEF2C-1 or si-MEF2C-2, downregulation of MEF2C was found in HUVECs, and the declined caused by si-MEF2C-1 was more pronounced, so si-MEF2C-1 was selected for the follow-up experiments (Figure 5C). Moreover, in HUA-treated HUVECs, let-7c expression was decreased by treatment with let-7c-inhibitor + si-NC or let-7c-inhibitor + si-MEF2C (Figure 5D). Meanwhile, there was increased MEF2C expression in HUA-treated HUVECs after treatment with oe-MEF2C or let-7c-inhibitor + si-NC (Figure 5E). RT-qPCR (Figure 5F), western blot analysis (Figure 5G) and ELISA (Figure 5H–5L) demonstrated that oe-MEF2C or let-7c-inhibitor alone reduced VCAM-1, ICAM-1, PAI-1 and TF expression and elevated T-PA expression in HUA- treated HUVECs. However, MEF2C silencing negated the effect of let-7c-inhibitor on VCAM-1, ICAM-1, T-PA, PAI-1 and TF expression. Furthermore, declined adhesion of monocytes to HUVECs was caused by oe-MEF2C or let-7c-inhibitor alone in HUA-treated HUVECs. The effects of let-7c-inhibitor were normalized by co-treatment with let-7c-inhibitor and si-MEF2C (Figure 5M). These results demonstrate that HUA increased let-7c expression to downregulate MEF2C, consequently inducing the expression of thrombus-related factors and the adhesion of monocytes and platelets to HUVECs.

### HUA-upregulated let-7c promotes activation of NF-κB pathway by repressing MEF2C

**Figure 6:**
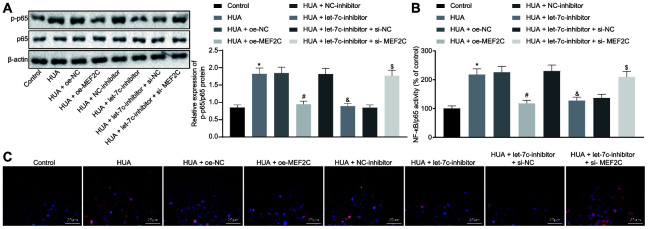
**HUA contributes to activation of the NF-κB pathway by repressing let-7c-targeted MEF2C.** Normal HUVECs were used as controls, and HUA-treated HUVECs were transfected or not transfected with oe-NC, oe-MEF2C, NC-inhibitor, let-7c-inhibitor, let-7c-inhibitor + si-NC, or let-7c-inhibitor + si-MEF2C. (**A**) p65 and phosphorylated p65 expression in HUVECs detected by western blot analysis normalized to β-actin. (**B**) NF-κB DNA activity in HUVECs detected by ELISA. (**C**) Phosphorylated p65 expression in HUVECs detected by immunofluorescence (× 400). * *p* < 0.05 *vs.* control HUVECs. # *p* < 0.05 *vs.* HUA HUVECs transfected with oe-NC. & *p* < 0.05 *vs.* HUA HUVECs transfected with NC-inhibitor. $ *p* < 0.05 *vs.* HUA HUVECs transfected with let-7c-inhibitor + si-NC. The measurement data were shown as mean ± standard deviation and compared by one-way analysis of variance, followed by Tukey's post hoc test. The cell experiment was repeated three times independently.

Next, we studied the downstream mechanism of MEF2C in thrombosis. Western blot analysis showed that p65 and phosphorylated p65 expression was enhanced in HUVECs following HUA treatment, and reduced after treatment with let-7c-inhibitor or oe-MEF2C. Silencing of MEF2C abrogated these effects of let-7c-inhibitor (Figure 6A). Meanwhile, ELISA analysis revealed that HUA treatment increased NF-κB DNA activity in HUVECs, but treatment with let-7c-inhibitor and oe-MEF2C triggered a decline of NF-κB DNA activity in HUA-treated HUVECs. Moreover, si-MEF2C negated the effects of let-7c-inhibitor on NF-κB DNA activity (Figure 6B). Immunofluorescence results in Figure 6C displayed the elevation of phosphorylated p65 expression in HUVECs following HUA treatment. Overexpression of MEF2C reduced phosphorylated p65 expression in HUA-treated HUVECs. Similarly, phosphorylated p65 expression in HUA-treated HUVECs was diminished after let-7c-inhibitor treatment, which was neutralized by silencing MEF2C. The above results suggest that HUA activated the NF-κB pathway by repressing MEF2C *via* let-7c upregulation.

### HUA activates NF-κB pathway to promote the expression of thrombus-related factors and the adhesion of monocytes and platelets to HUVECs

**Figure 7:**
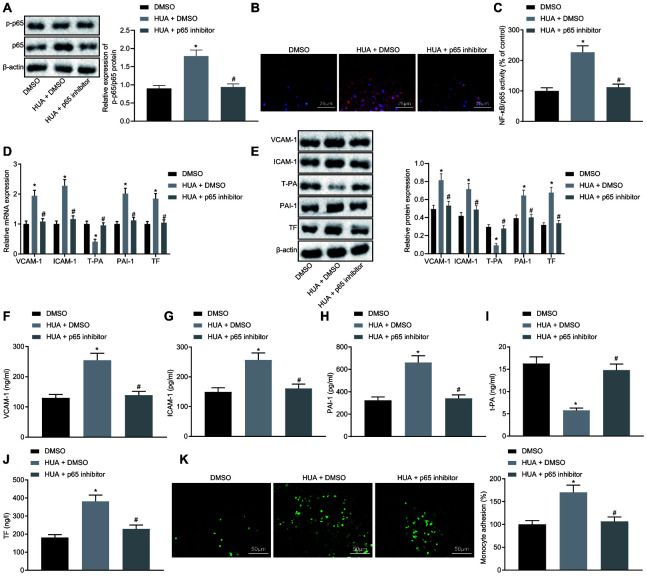
**HUA stimulates the expression of thrombus-related factors and the adhesion of monocytes and platelets to HUVECs *via* NF-κB pathway activation.** Normal HUVECs were treated DMSO, and HUA-treated HUVECs were treated with DMSO or p65 inhibitor. (**A**) p65 and phosphorylated p65 expression in HUVECs detected by western blot analysis normalized to β-actin. (**B**) Phosphorylated p65 expression in HUVECs detected by immunofluorescence (× 400). (**C**) NF-κB DNA activity in HUVECs detected by ELISA. (**D**) VCAM-1, ICAM-1, PAI-1, TF, and T-PA expression in HUVECs determined by RT-qPCR normalized to GAPDH. (**E**) VCAM-1, ICAM-1, PAI-1, TF, and T-PA expression in HUVECs determined by western blot analysis normalized to β-actin. (**F**) VCAM-1 level in HUVECs determined by ELISA. (**G**) ICAM-1 level in HUVECs determined by ELISA. (**H**) PAI-1 level in HUVECs determined by ELISA. (**I**) TF level in HUVECs determined by ELISA. (**J**) T-PA level in HUVECs determined by ELISA. (**K**) Adhesion of monocytes to HUVECs (× 200). * *p* < 0.05 *vs.* normal HUVECs treated with DMSO. # *p* < 0.05 *vs.* HUA HUVECs treated with DMSO. The measurement data were shown as mean ± standard deviation and compared by one-way analysis of variance, followed by Tukey's post hoc test. The cell experiment was repeated three times independently.

The above results indicated that HUA could inhibit MEF2C expression to activate NF-κB pathway by upregulating let-7c expression. To study whether HUA affects functions of HUVECs by activating NF-κB pathway, HUA-treated HUVECs were treated with p65 inhibitor. It was observed that p65 inhibitor resulted in decline in p65 and phosphorylated p65 expression and NF-κB DNA activity in HUA-treated HUVECs (Figure 7A–7C). Meanwhile, RT-qPCR (Figure 7D), western blot analysis (Figure 7E) and ELISA (Figure 7F–7J) documented reductions of VCAM-1, ICAM-1, PAI-1 and TF expression and increase of T-PA expression in HUA-treated HUVECs following p65 inhibitor treatment. Moreover, after HUA-treated HUVECs were treated with p65 inhibitor, there was reduced adhesion of monocytes to the HUVECs (Figure 7K). To sum up, the HUA-activated NF-κB pathway induces the expression of thrombus-related factors and the adhesion of monocytes and platelets to HUVECs.

## DISCUSSION

As a disease of localized blood clotting, thrombosis can occur in arterial or venous circulation, which has a significant medical impact [16]. Thrombosis results from abnormally activated platelets, which interact with endothelial cells, monocytes, erythrocytes, and neutrophils and then release fibrinogen, tissue factors as well as other agents [17]. HUA is associated with increased risk of cardiovascular events in patients with pre-existing cerebrovascular, diabetes and hypertension [18]. However, the specific mechanism of HUA underlying thrombosis still remained enigmatic. Therefore, we treated mice and HUVECs with HUA to explore the effect of HUA on thrombosis and its association with altered let-7c expression. Results of this study unveiled that HUA upregulated let-7c to activate MEF2C-dependent NF-κB pathway, thus inducing blood coagulation and thrombosis.

Initially results showed that HUA promoted blood coagulation and thrombosis, featured by decreased levels of APTT, PT and T-PA but increased TT, fibrinogen, D-dimer, VCAM-1, ICAM-1, PAI-1 and TF. As the final product of exogenous purine, UA is generally synthesized in liver, intestine and vascular endothelial cells, and is also endogenously generated from damaged, dying and dead cells, in which adenine, nucleic acid and guanine are degraded to uric acid [19]. Pervious study indicated that obese or hypertensive condition prolonged the thromboplastin time from 32.43 s to 44.18 s [20]. Furthermore, APTT and PT were prolonged when thrombus formation was decreased [21]. Besides, high levels of fibrinogen and D-dimer were observed in patients with cerebral venous sinus thrombosis [22]. Additionally, a study conducted by Folco et al. revealed that VCAM-1, ICAM-1 and TF levels were increased during the development of thrombosis [23] Meanwhile, thrombosis induced by lipopolysaccharide could be alleviated by inhibition of PAI-1 [24]. These results supported the notion that HUA promoted blood coagulation and thrombosis.

Moreover, a prior study reported that HUA triggered let-7c upregulation in endothelial cells [7], which is consistent with our results that let-7c was highly expressed in hyperuricemia patients and that HUA upregulated let-7c expression in mice. More importantly, we also found that let-7c upregulation induced blood coagulation and thrombosis, and the adhesion of monocytes to HUVECs *via* activation of NF-κB pathway by targeting MEF2C. On the contrary, certain studies indicated the inhibitory effect of let-7c on NF-κB as well. For example, let-7c attenuated the severity of pulpitis by inhibiting the NF-κB pathway in inflammatory disorders as NF-κB pathway increased the production of the inflammatory cytokines IL-1β and TNF-α and decreased stem cell viability [25]. Besides, let-7c was reported in other studies to inhibit and downregulate NF-κB phosphorylation [26–28]. However, let-7c did not directly regulate the NF-κB pathway, but targeted regulatory factors such as Ras and DMP1 to participate NF-κB pathway. Therefore, let-7c's effect on the NF-cB signaling pathway hinges on its target protein. Since let-7c's target protein MEF2 inhibited the NF-κB pathway, let-7c expression activated the NF-κB signaling pathway in this study.

Results from prior research showed decreased adhesion of monocytes and platelets to HUVECs to activate platelets and endothelial cells, consequently suppressing thrombosis [29]. Moreover, let-7c, as a platelet miR, had aberrant expression in platelets and influenced functions of platelets [9]. Similarly, let-7 overexpression promoted myocardial infarction in mice as induced by permanent ligation of left anterior descending coronary artery [10]. Furthermore, the targetscan prediction and dual luciferase reporter assay results in our study indicated MEF2C as a putative target of let-7c. It was elucidated that MEF2C alleviated myocardial ischemia by reducing coagulation [12]. Additionally, MEF2C inactivated NF-κB in inflammatory endothelial cells [13]. Inhibition of the NF-κB pathway caused by rivaroxaban relieved thrombosis in the DVT rat model [30]. Inactivation of NF-κB also can reduce coagulation by decreasing TF and PAI-1 levels in LPS-induced alveolar epithelial cell type II [31]. Bay 11-7082, a NF-κB inhibitor, decreased ICAM-1 and VCAM-1 expression in TNF-α-induced HUVECs to repress the adhesion of monocytes to TNF-α-treated HUVECs [32]. Hence, we conclude that let-7c stimulated thrombosis development by activating NF-κB pathway *via* MEF2C.

**Figure 8:**
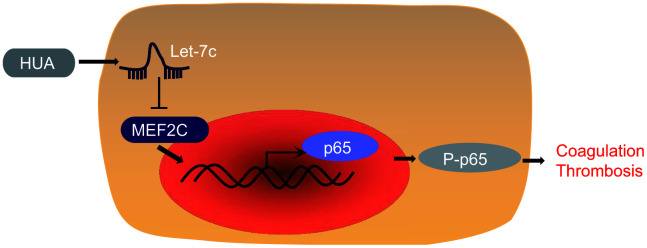
**The mechanism of HUA in thrombosis with involvement of let-7c and MEF2C.** HUA repressed MEF2C to activate NF-κB pathway by upregulating let-7c, ultimately causing blood coagulation and thrombosis.

In summary, our findings supported the pro-thrombotic effect of HUA. In brief, HUA downregulated MEF2C to activate NF-κB pathway by increasing let-7c expression, finally leading to thrombosis development (Figure 8). This discovery adds to our understanding of the complex mechanism of the HUA/let-7c/MEF2C/ NF-κB axis in thrombosis and provides a potential new therapeutic target for thrombosis. However, the present study has certain limitations given that HUVEC is a very specialized endothelial cell, so present findings may not generalize to other types of endothelial cells. This shall have to be established in future studies.

## MATERIALS AND METHODS

### Ethics approval and consent to participate

The ethics committee of the Affiliated Hospital of Qingdao University provided Ethical Approval for the experiments involved human beings in this study, which were guided by the specification of the *Declaration of Helsinki*. Approval was obtained from the donors or their relatives by written informed consent. The experiments involved animals were implemented in accordance with the principles embodied in the National Institutes of Health Guide for the Care and Use of Laboratory. Efforts were made to minimize animal use to avoid all unnecessary distress to the animals.

### Study subject

Totally 26 healthy adult males from the Affiliated Hospital of Qingdao University were enrolled as controls in this study, none of whom had hypertension, coronary heart disease, hyperglycemia, hyperuricemia, or complications with liver, kidney and other organs and systemic diseases after physical examination. In addition, 26 patients with hyperuricemia were enrolled in this study. The age, body mass index, blood pressure, blood glucose and blood lipid of healthy people were matched with those of patients with hyperuricemia. The diagnostic criteria were serum uric acid level of exceeding 420 μmol/mL (7 mg/dL). The effects of heart, liver, and renal insufficiency, as well as drugs, tumors, hematopathy and other secondary factors on blood uric acid were excluded. None of patients had hypertension, coronary heart disease, hyperglycemia, hyperlipidemia, cerebrovascular disease after physical examination. The 5 mL samples of fasting venous blood were collected and centrifuged at 3000 rpm for 10 min to obtain serum. After measurement of let-7c expression, the serum was stored at -70 °C for further analyses.

### Cell model and transfection

Human umbilical vein endothelial cell (HUVEC) lines [HUVECs and THP-1 monocytes, both from the American Type Culture Collection (ATCC, Manassas, VA, USA)] were cultured in basic medium (Gibco, Rockville, MD, USA) containing 10% fetal bovine serum (FBS), 100 mg/mL streptomycin and 100 U/mL penicillin at 37 °C with 5% CO_2_. Cells were grown in a 6-well plate until cell confluence reached approximately 80%. The medium was then replaced with serum-free medium and cell transfection was performed according to instructions in the Lipofectamine 2000 manual (Invitrogen, Inc., Carlsbad, CA, USA). HUVECs were treated with sodium hydroxide solution without addition of uric acid powder as blank control or dimethyl sulfoxide (DMSO). Additionally, other HUVECs were treated with high uric acid (HUA) [7]. After that, the HUA-treated HUVECs were further transfected with negative control (NC)-inhibitor, let-7c-inhibitor, overexpression (oe)-NC, oe-MEF2C, let-7c-inhibitor + small interfering (si)-NC, let-7c-inhibitor + si-MEF2C, DMSO, or NF-κB inhibitor JSH-23. The uric acid powder was solubilized in 1 mol/L sodium hydroxide solution at a concentration of 40 mmol/L. The uric acid was added into the medium to the final concentration of 600 μmol/L and the pH value of 7.2-7.4. The p65 inhibitor JSH-23 (J4455, Cat. No. 749886, Sigma-Aldrich, St Louis, MO, USA) was solubilized in DMSO and added into the medium at a final concentration of 50 μM to treat cells for 3 h [33]. The above plasmids were synthesized by Shanghai GenePharma Co, Ltd. (Shanghai, China), with sequences of let-7c-inhibitor: ACTCCATCATCCAACATACCAA and si-MEF2C: CAAUGUUGUCAGCAGUAUAGG.

### HUA mice model

A total of 40 C57BL/6J mice (aged 5-6 weeks, weighing 322-330 g, Medical Animal Laboratory, the Affiliated Hospital of Qingdao University) were utilized for establishment of hyperuricemia model [34]. In order to induce hyperuricemia, some mice were fed with normal food as controls, while the other mice were continuously fed with high yeast food (containing 10% yeast) for 3 weeks. NC inhibitor and let-7c-inhibitor sequences were embedded with lentivirus (Shanghai Gene Pharma Company) and the lentivirus was packaged in HEK293T cells (ATCC® CRL-11268™, ATCC cell bank). HEK293T cells were cultured in RPMI-1640 complete medium containing 10% FBS and passaged every other day. The lentivirus was diluted to 1 × 10^9^ pfu/100 μL with phosphate buffer saline (PBS). The 10 μL lentivirus was slowly injected into a tail vein with a 27-gauge needle daily for 3 days. Hyperuricemia mice were infected or not infected with lentivirus containing NC inhibitor and let-7c-inhibitor. After establishing the hyperuricemia model, the mice were anesthetized with 3% pentobarbital sodium (P3761, Sigma-Aldrich), followed by collection of 1 mL orbital blood. A 0.5 mL portion of blood was left at room temperature for 2 h, and centrifuged at 1000 rpm for 10 min to prepare serum. Serum uric acid (SUA) was detected using the phosphotungstic acid method with a Urea Assay Kit (MAK006, Sigma-Aldrich), followed by assessment of serum let-7c level. After anticoagulation of 0.5 mL venous blood with sodium citrate, samples were centrifuged at 10,000 rpm for 10 min to prepare plasma and to determine coagulation parameters. All plasma was stored at -70 °C before use.

### Platelet separation

Mouse platelets were extracted from the mouse blood samples as described in a previous literature [35]. Specifically, a blood collection tube containing K2-ethylenediaminetetraacetic acid was adopted to collect blood, which was centrifuged at 600 g and ambient temperature for 4 min. The top layer was platelet rich plasma. Platelet-rich plasma (Tianjin Blood Bank, Tianjin, China) was obtained from mice, supplemented with Prostaglandin E1 at a final concentration of 1 μM, and completely mixed. The liquid was removed following 10-min centrifugation of plasma at 800 g. Afterwards, platelet pellets were re-suspended in pre-warmed buffer (37 °C) containing 130 mM NaCl, 10 mM sodium citrate, 6 mM glucose, 9 mM NaHCO_3_, 0.9 mM MgCl_2_, 0.81 mM KH_2_PO_4_ and 10 mM Tris (pH 7.4).

### Reverse transcription quantitative polymerase chain reaction (RT-qPCR)

Total RNA was isolated from transfected HUVECs using TRIZOL (Invitrogen Inc.). The primers were designed and synthesized by Invitrogen Inc. (Table 2). The cDNA was then synthesized following instructions in the manuals provided by the TaqMan™ MicroRNA Reverse Transcription Kit (4366596, Thermo Fisher Scientific Inc., Waltham, MA, USA) and High-Capacity cDNA Reverse Transcription Kit (4368813, Thermo Fisher Scientific Inc.). RT-qPCR was conducted using the SYBR®Premix Ex TaqTM (Tli RNaseH Plus) Kit (RR820A, Takara Bio Inc., Tokyo, Japan) on the ABI7500 quantitative PCR instrument (Thermo Fisher Scientific Inc.). The relative expression level of mRNA or miR was normalized to glyceraldehyde-3-phosphate dehydrogenase (GAPDH) or U6 expression and was calculated using the 2^-ΔΔCt^ method [36].

**Table 2:** Primer sequences of related genes for RT-qPCR.

**Genes**	**Forward (5’-3’)**	**Reverse (5’-3’)**
let-7c	ACACTCCAGCTGGGTGAGGTAGTAGGTTGT	TGGTGTCGTGGAGTCG
MEF2C	GGTCACTGTAGGCATAGGA	TCATCTAGTGGCGACAAAGT
VCAM-1	ATCCCTACCATTGAAGATACTGG	TGATGACAGTTCTCCTTCTTTG
ICAM-1	ATCTGTGTCCCCCTCAAAAGTC	CCATCAGGGCAGTTTGAATAGC
t-PA	ATGCCCGATTCAGAAGAGG	GTTGAAACACCTTGGCTCGC
PAI-1	CTGGGTGAAGACACACACAAAAG	CACAGAGACAGTGCTGCCGT
TF	CTACTGTTTCAGTGTTCAAGCAGTGA	CAGTGCAATATAGCATTTGCAGTAGC
let-7c (mouse)	GGAAAGGACGAAACACCGGTATTTCTATCTACAACCTTGCCAAGC	TGTCTCGAGGTCGAGAATTAAAAAAGGTAATGCATTAAGGCCTC
U6 (mouse)	CGCTTCGGCAGCACATATAC	TTCACGAATTTGCGTGTCAT
U6 (human)	CTCGCTTCGGCAGCAGCACA	AACGCTTCACGAATTTGCGT
GAPDH (human)	ACCACAGTCCATGCCATCAC	TCCACCACCCTGTTGCTGTA

### Western blot analysis

Total protein was extracted from transfected HUVECs or mouse platelets using a radio-immunoprecipitation assay kit (R0010, Beijing Solarbio science & technology Co. Ltd., Beijing, China). Following addition of protease inhibitor (phenylmethylsulfonyl fluoride), a bicinchoninic acid protein assay kit (GBCBIO Technologies, Guangzhou, China) was employed to measure protein concentration. Samples containing 40 μg protein underwent separation by sodium dodecyl sulphate polyacrylamide gel electrophoresis. Then, the protein on the gel was electroblotted to a nitrocellulose membrane, which was sealed by tris-buffered saline with Tween 20 (TBST) containing 5% bovine serum albumin (BSA) at room temperature. The membrane was subsequently probed overnight at 4 °C with the diluted primary antibodies (Abcam, Cambridge, UK) to β-actin (ab8226, 1:1000, mouse), NF-κB p65 (ab16502, 1:1000, rabbit), phosphorylated NF-κB p65 (ab86299, 1:2000, rabbit), vascular cell adhesion molecule-1 (VCAM-1, ab134047, 1:2000, rabbit), intercellular adhesion molecular-1 (ICAM-1, ab134047, 1:2000, rabbit), plasminogen activator inhibitor (PAI) type 1 (PAI1, ab66705, 1:1000, rabbit), tissue plasminogen activator (t-PA, ab157469, 1:1000, rabbit), tissue factor (TF, ab104513, 1:1000, rabbit), MEF2C (ab211493, 1:1000, rabbit), CD41 (ab134131, 1: 2000, rabbit), and CD36 (ab13362, 1:1000, rabbit). Subsequently, the membrane was re-probed with horseradish peroxidase (HRP)-labeled secondary rabbit anti-mouse immunoglobulin G (IgG; ab6728, 1:1000, Abcam) and goat anti-rabbit IgG (ab150077, 1:1000, Abcam) at room temperature. The membrane was developed in developing liquid (NCI4106, Pierce, Rockford, IL, USA). The gray value of protein bands was analyzed by Image J gel image analysis software.

### Dual luciferase reporter assay

Artificially synthesized MEF2C 3'untranslated region (UTR) WT gene fragment was introduced into the pMIR-reporter (Huayueyang Biotechnology Co., Ltd., Beijing, China) using the endonuclease sites SpeI and Hind III. MUT (AUAAUC) sites of the complementary sequence of MEF2C 3'UTR-WT were designed and inserted using T4 DNA ligase into the pMIR-reporter. All these procedures were completed by Shanghai Genechem Co., Ltd. (Shanghai, China). Let-7c mimic and NC-mimic were independently co-transfected with correctly sequenced luciferase reporter plasmids for the WT and MUT into HEK293T cells for 48 h, followed by lysis. The luciferase activity was detected by a dual luciferase system and a Glomax20/20 luminometer, with the activity equal to the ratio between firefly luciferase and Renilla luciferase.

### NF-κB DNA-binding activity assay

Detection of NF-κB DNA binding activity was performed according to the manuals of the Nuclear Extract and Trans-Am NF-κB p65 ELISA Kits (Active Motif, Carlsbad, CA, USA). First, the nuclear and cytoplasmic components were prepared from the cells following directions in the Nuclear Extraction Kit. Nucleolytic proteins were added into a 96-well plate, which contained specific sequences bound by NF-κB p65 (5’-GGGACTTTCC-3’), followed by 1-h culture at ambient temperature for binding. The sample of each well was supplemented with a primary antibody specific for NF-κB, and then added with the second antibody coupled with HRP. An ELISA plate reader was utilized to detect the absorbance at 450 nm [37].

### Immunofluorescence

After fixing in PBS containing 4% formaldehyde for 30 min, HUVECs were permeated with 0.5% Triton X-100 for 30 min and sealed in 1% BSA for 30 min. Overnight culture of HUVECs was performed with primary rabbit NF-κB p65 antibody (1:500, ab86299) at 4 °C, followed by culture for 1 h in secondary goat anti-rabbit IgG H&L antibody (Alexa Fluor® 488, 1:500, ab150077, both from Abcam). The prepared samples were counterstained with 4'-6-diamidino-2-phenylindole for 10 min. A fluorescence microscope (Carl Zeiss, Jena, Germany) was employed to observe HUVECs [31].

### ELISA

Cell supernatant was obtained and stored at 80 °C, in which levels of T-PA, PAI-1, ICAM-1, TF and VCAM-1 were determined as per manufacturer's manuals for the ELISA Kit (Huamei Bio-company, Wuhan, China).

### Adhesion assay

Human THP-1 monocytes were cultured with 5,6-carboxyfluorescein diacetate succinimidyl ester (CFSE) solution (1 μM) at 37 °C for 30 min and washed in PBS to remove free CFSE in mixture. HUVECs were pretreated with 0.5 μg/mL NXT-EES, 1 μg/mL lipopolysaccharide (LPS) or NXT-EES + LPS overnight in a 24-well plate. On next day, the CFSE-labeled THP-1 monocytes (1 × 10^8^ cells/well) were supplemented into the HUVECs, followed by culture for 30 min at 37 °C. The non-adherent cells or platelets were removed by three PBS-washes. A Leica DMi8 microscope (Wetzlar, Germany) was employed to photograph the bound cells.

### Determination of coagulation parameters in mice

The Coag-Chrom 3003 Coagulometer and standard reagents (Bio-ksel, Poland) were applied to assess coagulation parameters including PT, APTT and TT levels in mouse plasma, following manufacturer’s protocols. Fibrinogen and D-dimer levels were evaluated using ELISA kits (69-20291, ABIN6200196, both from Wuhan Moshake Biotechnology Co., Ltd., Wuhan, China).

### Statistical analysis

All measurement data were shown as mean ± standard deviation and analyzed by SPSS 21.0 software (IBM, Armonk, NY, USA), with *p* < 0.05 as a level of statistically significance. If conforming to normal distribution and homogeneity of variance, unpaired data between two groups were compared by unpaired *t*-test, while comparisons among multiple groups were performed using one-way analysis of variance, followed by Tukey’s post hoc test.

### Ethics approval

The ethics committee of the Affiliated Hospital of Qingdao University provided Ethical Approval for the experiments involved human beings in this study, which were guided by the specification of the Declaration of Helsinki. Approval was obtained from the donors or their relatives by written informed consent. The experiments involved animals were implemented in accordance with the principles embodied in the National Institutes of Health Guide for the Care and Use of Laboratory. Efforts were made to minimize animal use to avoid all unnecessary distress to the animals.
